# Development and Pre-Clinical Evaluation of Two LAIV Strains against Potentially Pandemic H2N2 Influenza Virus

**DOI:** 10.1371/journal.pone.0102339

**Published:** 2014-07-24

**Authors:** Irina Isakova-Sivak, Jørgen de Jonge, Tatiana Smolonogina, Andrey Rekstin, Geert van Amerongen, Harry van Dijken, Justin Mouthaan, Paul Roholl, Victoria Kuznetsova, Elena Doroshenko, Vadim Tsvetnitsky, Larisa Rudenko

**Affiliations:** 1 Institute for Experimental Medicine, Saint Petersburg, Russia; 2 Centre for Infectious Disease Control, RIVM, Bilthoven, the Netherlands; 3 Animal Research Center, InTraVacc, Bilthoven, the Netherlands; 4 Microscope Consultancy, Weesp, the Netherlands; 5 PATH Vaccine Development Global Program, Seattle, Washington, United States of America; Boston University School of Medicine, United States of America

## Abstract

H2N2 Influenza A caused the Asian flu pandemic in 1957, circulated for more than 10 years and disappeared from the human population after 1968. Given that people born after 1968 are naïve to H2N2, that the virus still circulates in wild birds and that this influenza subtype has a proven pandemic track record, H2N2 is regarded as a potential pandemic threat. To prepare for an H2N2 pandemic, here we developed and tested in mice and ferrets two live attenuated influenza vaccines based on the haemagglutinins of the two different H2N2 lineages that circulated at the end of the cycle, using the well characterized A/Leningrad/134/17/57 (H2N2) master donor virus as the backbone. The vaccine strains containing the HA and NA of A/California/1/66 (clade 1) or A/Tokyo/3/67 (clade 2) showed a temperature sensitive and cold adapted phenotype and a reduced reproduction that was limited to the respiratory tract of mice, suggesting that the vaccines may be safe for use in humans. Both vaccine strains induced haemagglutination inhibition titers in mice. Vaccination abolished virus replication in the nose and lung and protected mice from weight loss after homologous and heterologous challenge with the respective donor wild type strains. In ferrets, the live attenuated vaccines induced high virus neutralizing, haemagglutination and neuraminidase inhibition titers, however; the vaccine based on the A/California/1/66 wt virus induced higher homologous and better cross-reactive antibody responses than the A/Tokyo/3/67 based vaccine. In line with this observation, was the higher virus reduction observed in the throat and nose of ferrets vaccinated with this vaccine after challenge with either of the wild type donor viruses. Moreover, both vaccines clearly reduced the infection-induced rhinitis observed in placebo-vaccinated ferrets. The results favor the vaccine based on the A/California/1/66 isolate, which will be evaluated in a clinical study.

## Introduction

Seasonal epidemics of influenza virus annually cause significant disease burden [1]. On top of that, pandemics caused by influenza variants to which the population was naïve occurred four times during the last century. These were characterized by very rapid spread, affected entire continents or the whole world with morbidity rates considerably above normal and excess mortality in some population groups. This highlights the seriousness of the threat to mankind that lies in possible future pandemics.

To prepare for future pandemics, a plan for clinical testing of pandemic vaccines was drawn up and a classification of candidate pandemic vaccine priority was proposed at the international conference on pandemic influenza vaccines in 2003. The virus subtypes H1, H2, and H3 which are known to have caused previous pandemics have the highest level, and H5, H6, H7, and H9 have high level of priority [2].

In particular, special attention should be focused on influenza viruses that previously circulated in the human population but disappeared from circulation for a long time, resulting in lack of immunity in a large part of the population. Subtype H2N2 influenza viruses are an especially stark example of this situation. H2N2 influenza viruses have not circulated in the human population since 1968, so people born after this year have no immunity to them and will therefore be vulnerable to this virus if it returns to circulation [3], [4]. According to sero-archeological data, this subtype caused the 1889 pandemic and circulated until 1901, after which it was displaced by another virus subtype [5]. However, 56 years later, H2N2 viruses returned to circulation in 1957, causing the worldwide “Asian flu” pandemic that took over two million lives [5], [6]. H2N2 influenza viruses continue to circulate in the avian reservoir, emphasizing the likelihood for their return to the human population. Therefore leading virologists are recommending an H2N2 vaccination campaign to be initiated now, before a pandemic breaks out [3], [7]–[11].

Russian master donor virus A/Leningrad/134/17/57 (H2N2) (Len/17) for type A live attenuated influenza vaccine (LAIV) can potentially be used as an H2N2 vaccine strain. This virus was used back in the 1960s as a vaccine to immunize children and adults [12]. However, during the 1957–1968 virus circulation period, the H2N2 strain underwent serious evolutionary changes, and the immune response to the early H2N2 viruses may be ineffective against viruses that circulated at the end of the H2N2 wave [13]. In addition, H2N2 viruses which circulated at the end of H2N2 inter pandemic period diverged into two lineages with substantially distinct antigenic properties [13]. Since it is impossible to predict antigenic properties of the viruses in case of a new H2N2 pandemic, we used classical reassortment technique to prepare two H2N2 LAIV strains containing HA and NA surface antigens from either A/California/1/66 (Clade I) or A/Tokyo/3/67 (Clade II) human H2N2 influenza viruses. Both vaccine strains were studied *in vitro* and *in vivo* to demonstrate their attenuation, immunogenicity, cross–reactivity and cross–protection in order to select candidates for future clinical trials.

## Materials and Methods

### Ethics statement

6–8 week old female CBA mice were purchased the Laboratory Animal Farm “RAPPOLOVO” (Rappolovo, North–West region, Russia), and kept with unlimited access to food and water. All procedures were performed under ether anesthesia. The Mouse study was approved by the Institute of Experimental Medicine Animal Research Ethics Board. All animal work was conducted in accordance to national and international guidelines to minimize discomfort to animals.

The protocol for the experiment in ferrets was approved by the independent Animal Experimental Committee of the Netherlands Vaccine Institute (Bilthoven, the Netherlands) under permit number 201100328 and was performed in BSL-3 isolators. Animal handling was conducted in accordance with relevant Dutch national legislation, including the 1997 Dutch Act on Animal Experimentation. Animals were examined for general health on a daily basis and experimental animal handling was performed using a light anestheticum (ketamine 5 mg/kg and medetomidine hydrochloride (0.1 mg/kg mixture). After handling, the anesthesia was antagonized with atipamezole (0.25 mg/kg) with the exception that after intranasal vaccination and challenge, ferrets were not antagonized to avoid a sneezing reflex. All animals had *ad libitum* access to pelleted standard ferret food and tap water.

### 2.1 Viruses

Cold-adapted A/Leningrad/134/17/57 (H2N2) master donor virus (MDV), as well as wild-type strains A/Leningrad/134/57 (H2N2) and A/New Caledonia/20/99 (H1N1), were obtained from the influenza strain repository of the Institute of Experimental Medicine (St. Petersburg, Russia). A/17/duck/Potsdam/86/92 (H5N2) reassortant, which contains HA gene of A/duck/Potsdam/1402-6/86 (H5N2) avian influenza virus and six internal genes and the N2 gene of A/Leningrad/134/1957 (H2N2) was also obtained from the repository of IEM. Wild-type A/California/1/66 (H2N2) and A/Tokyo/3/67 (H2N2) viruses were from the CDC repository (Atlanta, GA, USA). HK-X31, a PR8 based reassortant containing the HA and NA genes of A/Hong Kong/1/1968 was obtained from the repository of the RIVM. For mouse studies and all mouse related *in vitro* assays viruses were propagated in embryonated chicken eggs at permissive temperatures (33°C for cold-adapted virus and 37°C for wild-type viruses). Virus-containing allantoic fluid was clarified by low-speed centrifugation and virus aliquots were stored at −70°C until use. For ferret studies and all ferret related *in vitro* assays, viruses were propagated on MDCK cells and clarified by low-speed centrifugation and aliquots were stored at −70°C. Infectious virus titers were either determined by limited dilutions in eggs or by end-point titration on MDCK cells. Virus titers were calculated by the method of Reed and Muench [14] and expressed as log_10_EID_50_/ml or log_10_TCID_50_/ml.

### 2.2 Generation of reassortant viruses

A novel strategy was developed to prepare reassortant viruses between H2N2 wild-type viruses and Len/17 master donor virus which has the same H2N2 subtype. Our initial attempt to generate reassortants by direct co-infection was not successful since immune serum directed against Len/17 surface antigens cross-reacted with HA and NA of wild-type viruses, and as a result, no viable virus was recovered after selective passages. To overcome this issue we first generated an intermediate reassortant strain A/17/New Caledonia/99/513 (17/NC/513) bearing HA and NA genes of A/New Caledonia/20/99 (H1N1) virus and six internal genes of Len/17 MDV. This intermediate virus was further used as a source of Len/17-specific internal genes. Thus, H2N2 LAIV reassortant viruses were generated by a well-established method of classical reassortment in chicken embryos, except that 17/NC/513 strain served as master donor virus [15]. Briefly, parental viruses – either A/California/1/66 (H2N2) or A/Tokyo/3/67 (H2N2) and MDV 17/NC/513 - were mixed in equal doses 10^6^ EID_50_ and administered together into embryonated eggs followed by 48 hours incubation at optimal temperature. The resulting mixture of reassortants was subjected to several cloning passages by limiting dilutions at low temperature 26°C in the presence of hyper immune serum against MDV. Viruses derived after this cloning procedure were subsequently cloned by two additional cloning procedures at optimal temperature without antiserum. The genome composition of multiple resulting reassortant viruses was determined by partial sequencing of all eight viral genes. To this end, we used universal primer pairs which were able to react with genes of all known subtypes of human influenza viruses, and these primers allowed sequencing of short regions of 300–500 nucleotides which, in turn, was sufficient to determine gene origin of any reassortant [16] (Table 1). After the reassortants with required genome composition were selected, namely A/17/California/66/395 (17/Cal/395) and A/17/Tokyo/67/326 (17/Tok/326), they were further subjected to phenotypic analyses and full-genome sequencing.

**Table 1:** List of universal primers, which were used for partial sequencing of genes of H2N2 reassortant influenza viruses.

#	Gene	Start position	Sequence	Reference
**1**	PB2	F1757	TGGAATTTGARCCATTT	this study
**2**	PB2	R2341	ACTGGCTCTTCTATTAGTAGAAACAAGGTCGTTT	[16]
**3**	PB1	F1	GATCGCTCTTCAGGGAGCGAAAGCAGGCA	[16]
**4**	PB1	R1078	TGCCATTTTRTTTGAGAACATTAT	this study
**5**	PA	F1	GATCGCTCTTCAGGGAGCGAAAGCAGGTAC	[16]
**6**	PA	R619	CTTCGCCTCTTTCGGACTGACG	this study
**7**	HA	F1	GATCGCTCTTCAGGGAGCAAAAGCAGGGG	[16]
**8**	HA	R1776	ACTGGCTCTTCTATTAGTAGAAACAAGGGTGTTTT	[16]
**9**	NP	F1	GATCGCTCTTCAGGGAGCAAAAGCAGGGTA	[16]
**10**	NP	R1222	CACTYCTGGTYCTTATGG	this study
**11**	NA	F1	GATCGCTCTTCAGGGAGCAAAAGCAGGAGT	[16]
**12**	NA	R1466	ACTGGCTCTTCTATTAGTAGAAACAAGGAGTTTTTT	[16]
**13**	M	F1	GATCGCTCTTCAGGGAGCAAAAGCAGGTAG	[16]
**14**	M	R1027	ACTGGCTCTTCTATTAGTAGAAACAAGGTAGTTTTT	[16]
**15**	NS	F1	GATCGCTCTTCAGGGAGCAAAAGCAGGGTG	[16]
**16**	NS	R890	ACTGGCTCTTCTATTAGTAGAAACAAGGGTGTTTTT	[16]

### 2.3 Phenotypic and genetic analyses

The temperature-sensitive (*ts*) and cold-adapted (*ca*) phenotypes of parental and reassortant viruses were assessed by evaluating viral replication in eggs at permissive (33°C) and restrictive temperatures (26, 37, 38, 39 and 40°C). Eggs were incubated for 48 hours for temperatures 33–40°C and 6 days for 26°C before the detection of virus by hemagglutination assay.

Genetic analysis of reassortant viruses was performed either by partial or full-genome sequencing using a BigDye Terminator v3.1 cycle sequencing kit (Applied Biosystems) and a 3130xl Genetic Analyzer (Applied Biosystems) according to the instructions of the manufacturer.

### 2.4 Virus replication in mice and median mouse intranasal infectious dose (MID_50_) determination of H2N2 LAIVs

Groups of 5 female CBA mice were inoculated with a volume of 50 µl of virus suspension containing the appropriate infectivity dose of corresponding virus (10^1^ to10^6^ EID_50_) by the intranasal (i.n.) route. Mouse lungs were collected at 3 days post infection (dpi) and stored frozen at −70°C until used for homogenization. Tissue homogenates were prepared using a small bead mill (QiagenTissueLyser LT) in 1 ml of sterile PBS and the clarified by low-speed centrifugation supernatants were inoculated into eggs to determine the presence of infectious virus, as an indicator of mouse infection. The MID_50_ was calculated as the median EID_50_ dose required to infect 50% of the animals. To study attenuated phenotype of H2N2 LAIV strains groups of 6 female CBA mice were infected i.n. with the corresponding viruses at a dose of 100 MID_50_ in a volume of 50 µl. Three days after inoculation, mice were euthanized and lungs, nasal turbinated and brains were harvested. Virus titers in the organs were determined by end-point titration in eggs and expressed as log_10_EID_50_/ml.

### 2.5 Immunogenicity and protective efficacy of H2N2 LAIV candidates in a mouse model

Groups of 18 female CBA mice were inoculated i.n. with either 100 or 1000 MID_50_ of each H2N2 LAIV (17/Cal/395 and 17/Tok/326) in a volume of 50 µl. Two doses of the same virus were administered 21 days apart. Control mice received PBS. Blood samples were collected prior immunization, 21 days after first dose and 21 days after second dose of the vaccine. To remove temperature-stable inhibitors, the sera were treated with RDE (Receptor destroying enzyme, Denka-Seiken, Japan). Serum antibody titers were determined by haemagglutination inhibition (HI) test using four haemagglutinating units of virus and a 0.5% suspension of chicken red blood cells. To study the vaccine's protective efficacy, 9 animals in each group were challenged with 100MID_50_ of homologous H2N2 wt virus, and 9 remaining animals with 100 MID_50_ of heterologous wt virus on day 42 after the first immunization. Three days after the challenge, respiratory (lung and nasal turbinates) and systemic (spleen and brain) organs were collected from four mice in each group. Harvested organs were stored at −70°C until titration. Virus titers in organ homogenates were determined as described above. The remaining five animals in each group were weighed and monitored daily until Day14 after the challenge.

### 2.6 Immunogenicity and protective efficacy of H2N2 LAIV candidates in a ferret model

Female Ferrets (*Mustela putorius furo*) of approximately 12 months old were selected based on a negative test for previous influenza and Aleutian disease infections. The animals were implanted with a temperature logger (DST micro T, Star-Oddi) in the peritoneal cavity 14 days prior to immunization. The temperature was recorded every 30 minutes. The animals (48 in total) were allocated to 4 groups of 12 ferrets and were intranasally vaccinated with a single dosage of either the 17/Cal/395 or 17/Tok/326 H2N2 vaccine or the control MDV Len/17, using a dose of 10^6^ TCID_50_ in a volume of 0.5 ml. Control animals received a placebo (PBS). After vaccination the animals were housed in isolators (6 animals per isolator) to overcome cross infection of the different LAIV which are potentially shedded by the vaccinated groups. Blood samples were collected one day before immunization and 14, 21 and 26 days after vaccination for serological analysis (HI, VN and NI assays). Twenty-one days after vaccination, six ferrets from each group were challenged with A/California/1/66 wild-type virus, and the remaining 6 animals were challenged with A/Tokyo/3/67 virus. Challenge viruses were administered intranasally at a dose of 10^6^ TCID_50_ in a volume of 0.5 ml and the ferrets within a group receiving a different challenge virus were kept in separate isolators. From day 21 until the end of the study, ferrets were monitored for clinical disease. During the challenge phase, animals were weighed and throat swabs were taken on day 21, 23, 24 and 26. Swabs were vortexed in 2 ml transport buffer (15% sucrose, 2.5 µg/mL Fungizone, 100 U/mL Penicillin, 100 µg/mL Streptomycin, 250 µg/mL Gentamicin in PBS) and stored at −70°C until use. At the day of termination (day 26) ferrets were sacrificed by bleeding and the nasal turbinates were isolated for virus titer determination and histopathology and the trachea and lung for virus titer determination only. Organs were homogenized using a FastPrep-24 (MP Biomedicals) homogenizer and clarified by low speed centrifugation. The infectious viral titers in throat swabs and organs were determined by end-point titration in MDCK cells using a 5 fold dilution strategy and expressed as log_10_TCID_50_/ml. Body temperature was analyzed by the Area Under Curve (AUC) of the progression in time using the trapezoidal rule.

### 2.7 Histopathological analysis

Nasal turbinates were fixed in 10% buffered formalin slightly decalcified and embedded in paraffin. Paraffin sections were cut at a thickness of 5 µm and stained with haematoxylin and eosin. Slides were examined microscopically and the infection damage was evaluated using the following histopathological parameters: damage to the epithelial linings, presence of inflammatory cells, and the presence of exudate and/or hemorrhages. These parameters were semi-quantitatively scored on a scale of 0 (no abberations) to 5 (severe damage). The end score was determined by taking into account the severity and percentage of tissue affected of the various parameters.

### 2.8 Virus Neutralization assay

Virus neutralizing titers in ferrets sera were determined as described elsewhere [17]. Briefly, sera were heat inactivated for 30 minutes at 56°C and two fold serially diluted in virus growth medium (MEM medium (Gibco, 31095) containing: 40 µg/ml gentamycin (Sigma), 0.01 M Tricin (Sigma) and 2 µg/ml TPCK treated trypsin (Sigma)) in a 96 wells plate using a starting dilution of 1∶8. An equal volume of virus at a concentration of 100 TCID_50_ was added to each well. Wells containing only medium or medium with only virus served as a cell viability and negative control respectively. In addition, a back titration of the virus stock used for incubation with the serum dilutions was prepared by ½log10 serial dilutions. All the preparations were subsequently incubated for 2 hours at 37°C. Following the virus-serum mixture, the controls and the back-titration samples were transferred to 96 wells plates containing a confluent monolayer of MDCK cells and incubated for another 2 hours at 37°C, 5% CO_2_ after which the medium was refreshed. Plates were incubated until the back titration plate reached CPE at a titer of 100 TCID_50_, which usually occurred after 4–5 days.

### 2.9 Neuraminidase activity of H2N2 LAIV reassortant viruses

NA activity of the H2N2 strains was measured as described previously [18]. Briefly, viruses were purified from the allantoic fluid by sedimentation followed by ultracentrifugation on a 30–60% sucrose step gradient. 96-well plates (Sarstedt, Germany) were coated overnight with 150 µl of 50 µg/ml fetuin (Sigma-Aldrich, USA). Serial 2-fold dilutions of purified viruses were prepared in PBS containing 1% bovine serum albumin (BSA) to obtain virus concentrations between 8 AU and 512 AU. 100 µl of pre-diluted virus series were transferred to the fetuin-coated wells. As a positive control one row contained wells with RDE (Denka Seiken) diluted 1∶10 in PBS-BSA. After 1 h incubation at 37°C the plates were washed, supplemented with 100 µl of peroxidase-labeled peanut lectin (2.5 µg/ml; Sigma-Aldrich, USA) and incubated for 1 h at room temperature and washed again. One hundred µl of peroxidase substrate (TMB) was added and the reaction was stopped after 5 min with 100 µl of 1 N sulfuric acid. NA activity was calculated from OD values measured at 450 nm using the universal microplate reader (ELx800, Bio-Tek Instruments Inc, USA).

### 2.10 Neuraminidase Inhibition antibody titers

Two fold serial dilutions of serum were prepared in duplicates and incubated with a virus dilution of which the neuraminidase activity results in an OD of 1 for 1 hour at 37°C. Following, this mixture was transferred to overnight coated fetuin plates and the assay was further performed as described above to measure the neuraminidase activity. The NI titer was determined from the last dilution equal to or below 50% of the Neuraminidase activity of the virus control.

### 2.11 Statistical analyses

Data were analyzed with the Statistica software (version 6.0; Statsoft Inc. or GraphPad Prism 6.03). Virus titers and antibody titers were log transformed and significant differences were determined by the Mann-Whitney U-test. The pathology scores were analyzed by an adapted version of the Wilcoxon-Mann-Whitney test using mid p-values validated for original data with a small sample size. *P* values were corrected for multiple comparisons using the Benjamini-Hochberg method. *P* values of <0.05 were considered significant.

## Results

### 3.1 Generation of H2N2 LAIV reassortants

For our study we selected two wild-type viruses A/Tokyo/3/67 (H2N2) (A/Tok/3/67) and A/California/1/66 (H2N2) (A/Cal/1/66), which belong to two divergent lineages that circulated at the end of H2N2 wave [13]. Internal genes of MDV A/Leningrad/134/17/57 were transferred to H2N2 vaccine reassortants from an intermediate reassortant strain A/17/New Caledonia/99/513 (H1N1) (17/NC) bearing HA and NA genes of A/New Caledonia/20/99 (H1N1) and six internal genes of Len/17 MDV. The resulting vaccine H2N2 reassortant viruses A/17/California/66/395 (17/Cal/395) and A/17/Tokyo/67/326 (17/Tok/326) inherited six internal genes from Len/17 MDV and HA and NA genes from A/Cal/1/66 and A/Tok/3/67 viruses, respectively (Table 1). Full-genome sequencing of both H2N2 LAIV reassortants confirmed sequence identity with all parental genes with an exception of one amino acid change Glu-121-Gly (H2 numbering) noted in HA1 protein of 17/Tok/326 strain compared to wild-type parental virus A/Tok/3/67. It is worth mentioning that A/Cal/1/66 as well as 17/Cal/395 viruses have Phe at position 226 of HA1 receptor-binding site (H3 numbering), whereas A/Tok/3/67 and 17/Tok/326 strains have Leu at this position. The latter of which is typical for viruses with preferential binding of α2,6 human type receptors [19].

### 3.2 Phenotypic study of H2N2 LAIV reassortants

Phenotypic properties of H2N2 LAIV vaccine candidates were determined by virus titration in embryonated chicken eggs at various temperatures. As shown in Table 2, both vaccine candidates resembled temperature-sensitive and cold-adapted phenotype of MDV Len/17 and, importantly, of reassortant virus 17/NC/513 used as a source of Len/17 internal genes. Thus, vaccine strains grew poorly at temperatures of 38°C and above but their reproduction activity at low temperature of 26°C was quite efficient. In contrast, wild-type virus A/Tok/3/67 expressed *non-ts* phenotype at 38°C and 39°C and was unable to grow at low temperatures. Interestingly, wild-type virus A/Cal/1/66 was temperature-sensitive at 39°C but grew efficiently at 38°C. Therefore, both H2N2 vaccine candidates 17/Cal/395 and 17/Tok/326 were demonstrated to possess clear *ts* and *ca* phenotype which are required for LAIV candidates.

**Table 2:** Reproductive capacity at different temperatures in eggs and median mouse infection dose (MID_50_) of H2N2 LAIV candidates and their parental viruses.

Virus	Infectious viral titer at indicated temperature, log_10_EID_50_/ml					Phenotype	MID_50_, log_10_EID_50_
	33°C	38°C	39°C	40°C	26°C		
Len/17 (H2N2)	9.2±0.4	3.2±0.5	2.2±0.2	≤1.2	6.7±0.2	*ts, ca*	4.5
17/NC (H1N1)	8.9±0.5	3.2±0.5	1.8±0.5	n/t	7.0±0.2	*ts, ca*	4.7
A/Cal/1/66 (H2N2)	8.0±0.6	7.1±0.5	2.4±0.5	2.2±0.4	2.8±0.8	*±ts, non-ca*	1.0
A/Tok/3/67 (H2N2)	7.2±0.3	6.7±0.7	6.7±0.5	3.0±0.3	2.1±1.2	*non-ts, non-ca*	1.0
17/Cal/395 (H2N2)	9.0±0.3	3.1±0.8	3.2±0.5	n/t	6.6±0.7	*ts, ca*	4.3
17/Tok/326 (H2N2)	9.2±0.7	3.5±0.7	3.2±0.5	n/t	6.3±0.8	*ts, ca*	4.9

n/t, not tested.

### 3.3 Antigenic characterization of H2N2 viruses

Antigenic properties of H2N2 viruses were studied in haemagglutination inhibition assays using hyper-immune rat sera raised against all these viruses. As demonstrated in Table 3, H2N2 LAIV strains were antigenically identical to their parental viruses: 17/Cal/395 vaccine candidate was neutralized by anti-A/Cal/1/66 serum to the same extent as by homologous antiserum, and vice versa. The same results obtained for 17/Tok/326 LAIV candidate and A/Tok/3/67 wild-type parental virus provided evidence that single mutation Glu-121-Gly in HA1 protein has negligible, if any impact on virus antigenic properties. Interestingly, antibodies raised to A/Cal/1/66 virus were strongly strain-specific and did not cross-react with either Len/17 or A/Tok/3/67 virus, whereas anti-Len/17 antibodies reacted to the same titer with A/Cal/1/66 virus and, to a lower extent, with A/Tok/3/67 virus. Antiserum raised against A/Tok/3/67 cross-reacted with A/Cal/1/66 virus to 1/8 of homologous titer, but no cross-reaction was noted with Len/17 MDV. Altogether, anti-Len/17 immune serum demonstrated broad cross-reactivity to H2N2 viruses that appeared later in the circulation, whereas sera to later viruses were unable to neutralize earlier H2N2 strain.

**Table 3:** Antigenic characterization of H2N2 viruses.

Virus	Reciprocal HI-titer with hyper-immune rat serum raised against:				
	Len/17	Cal-wt	17/Cal/395	Tok-wt	17/Tok/326
Len/17	160	5	5	5	5
A/Cal/1/66	160	2560	2560	40	40
17/Cal/395	160	2560	2560	20	20
A/Tok/3/67	20	5	5	320	320
17/Tok/326	10	5	5	320	320

### 3.4 Pre-clinical studies of H2N2 LAIV reassortants

#### 3.4.1 Safety and attenuation phenotype of H2N2 LAIV reassortants in a mouse model

Median mouse infection dose of H2N2 LAIV candidates and their wild-type parental viruses was determined by virus detection in mouse lungs three days after intranasal infection. As shown in Table 2, wild-type viruses easily infected the lower respiratory tract of mice and the MID_50_ value did not exceed 1.0 log_10_, whereas 4.5 to 4.9 log_10_ were required for cold-adapted viruses to infect 50% of the mice. These data are in concordance with previous findings about restriction of *ca* virus replication in mouse lung [20], [21].

**Figure 1:**
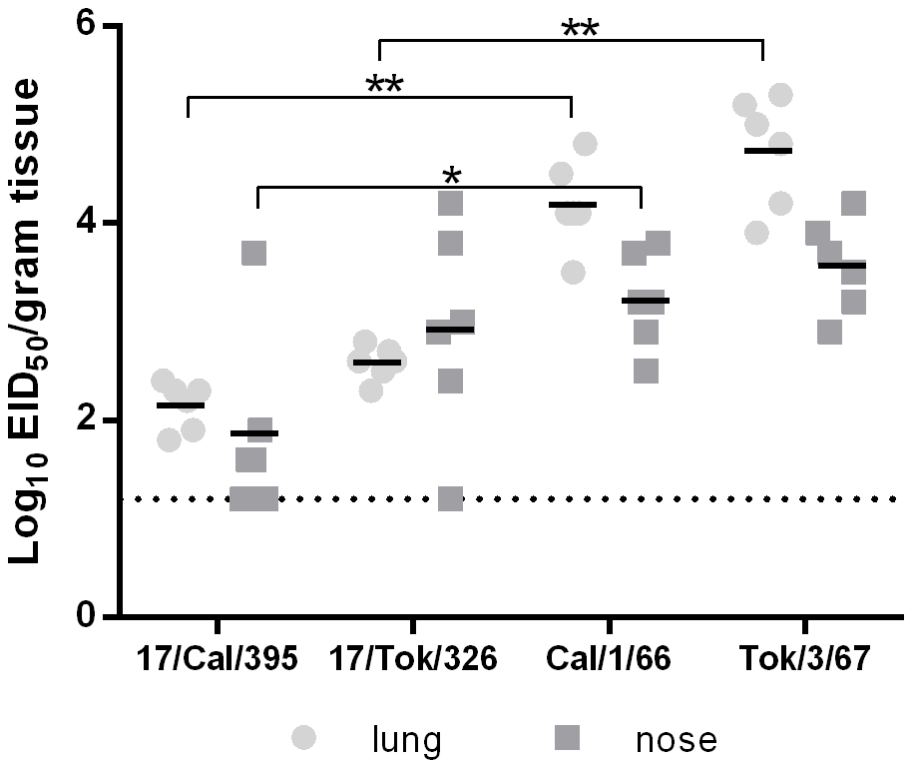
Replication of H2N2 influenza viruses in respiratory organs and brain of mice. Groups of 6 female CBA mice were infected i.n. with the corresponding viruses at a dose of 100 MID_50_ in a volume of 50 µl. Three days after inoculation, mice were euthanized and lungs and nasal turbinateswere harvested. Virus titers in the organs were determined by end-point titration in eggs and expressed as log_10_EID_50_/ml. Significant lower titers with respect to the donor viruses are indicated by * (p<0,05) or ** (p<0,005). Bars indicate the average titer.

To evaluate the safety of H2N2 LAIV candidates with respect to reproduction in the lower respiratory tract and in the brain, groups of mice were infected i.n. with 100 MID_50_ of vaccine candidates, or in parallel with their wild-type parental viruses. As shown in Figure 1, H2N2 LAIV candidates were able to reproduce in the upper and, to a limited extent, in the lower respiratory tract of mice. The levels of reproduction of H2N2 vaccine candidates averaged 1.8–2.7 log_10_ in the nasal turbinates and 2.2–2.5 log_10_ in the lungs. In contrast, the wild-type strains A/Cal/1/66 and A/Tok/3/67 reproduced actively both in the nasal turbinates (3.2–3.5 log_10_), and the lungs (4.2–4.7 log_10_) of mice. Importantly, neither H2N2 LAIV candidates, nor parent viruses A/Cal/1/66 and A/Tok/3/67 were isolated from the brains (not shown) of infected animals, indicating the lack of neuroinvasiveness of these viruses (Figure 1). The production profile characteristic for cold-adapted attenuated influenza viruses and the absence of neuroinvasion after intranasal administration suggest an acceptable safety profile for the use of H2N2 LAIV reassortants in humans.

#### 3.4.2 Immunogenicity and protective efficacy of H2N2 LAIV reassortants in a mouse model

**Figure 2:**
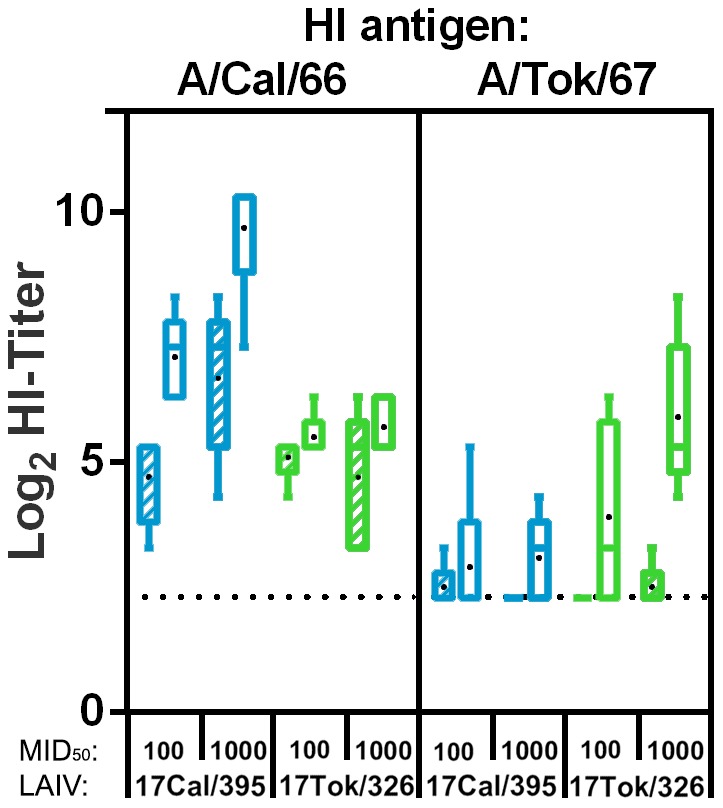
Immunogenicity and cross-reactivity of H2N2 LAIV candidates in a mouse model. Homologous and cross-reactive antibody responses in mice 21 days after 1^st^ vaccination (hatched boxes) and 21 days after 2^nd^ vaccination (day 42; open boxes) as measured by haemagglutination inhibition. Titers induced by the 17/Cal/395 (blue) and 17/Tok/326 (green) vaccines (either at a dose of 100 MID_50_ or 1000 MID_50_) are grouped by the respective antigens indicated at the top of the panels. Serum was titrated against 4HAU of the respective wild type viruses using 0.5% chicken erythrocytes. In sera of placebo vaccinated animals and sera prior to vaccination no titers above detection limit were detected (not shown). Presented are box plots of log_2_ transformed HI-titers. Bars indicate the 5–95 percentile and black dots indicate the average titer.

We evaluated the immunogenicity and protective efficacy of two different doses of each H2N2 LAIV candidate: 100 MID_50_ and 1000 MID_50_, each delivered 21 days apart. Humoral immune responses to both vaccine strains were analyzed 21 days after each vaccination. As shown in Figure 2, 17/Cal/395 LAIV candidate was more immunogenic than 17/Tok/326 virus and was able to elicit high homologous HI antibody titers after two doses (7.1 and 9.7 log_2_ with 100 MID_50_ and 1000 MID_50_, respectively), and even after a single immunization with the high dose (6.7 log_2_, Figure 2, left panel). In contrast, 17/Tok/326 LAIV candidate failed to induce homologous HI antibody immune response after a single dose even using the high dose. Two doses of 1000 MID_50_ of this vaccine were required to elicit levels of homologous antibody titer (5.9 log_2_, Figure 2, right panel) which are considered to be protective in mice. Interestingly, immunization with 17/Tok/326 induced cross-reactive HI antibodies to A/Cal/1/66 wild-type virus at levels of 4.7–5.5 log_2_, whereas almost no cross-reactivity was noted in sera of 17/Cal/395-immunized mice.

**Figure 3:**
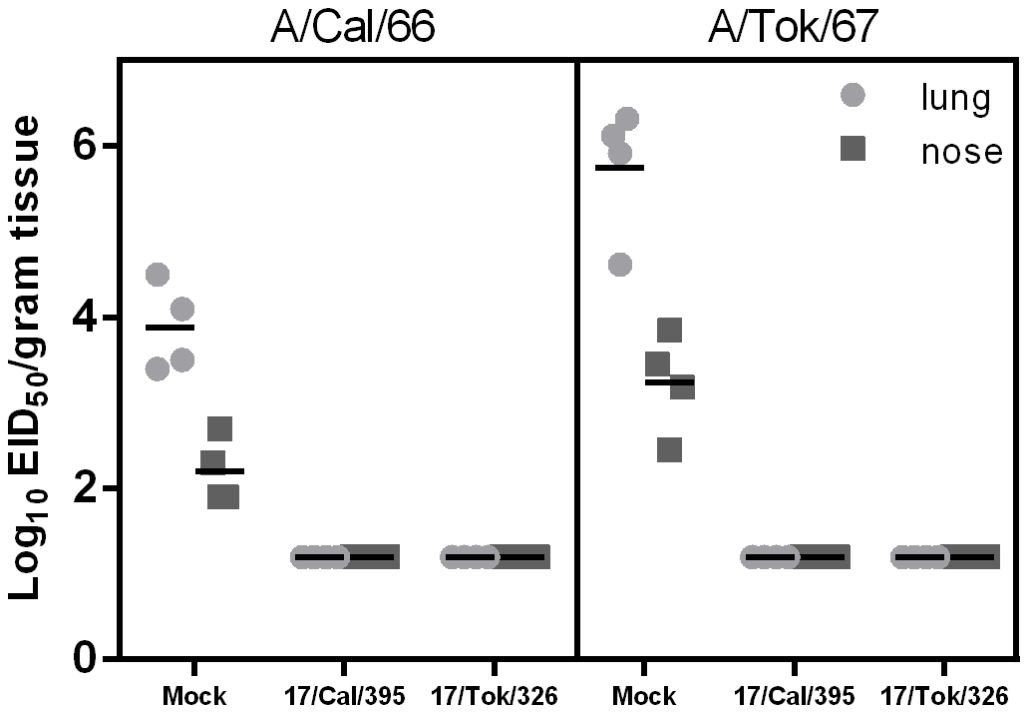
Replication of H2N2 challenge viruses in organs of immunized mice. The animals were challenged with 100 MID_50_ of either A/California/1/66 (left panel) or A/Tokyo/3/67 (right panel) wild-type virus on day 42 after the first immunization. Three days after challenge, respiratory (lung and nasal turbinates) and systemic (spleen and brain – not shown) organs were collected from four mice in each group. Virus titers in the organs were determined by end-point titration in eggs and expressed as log_10_EID_50_/ml. The depicted vaccine groups represent the low (100MID_50_) and high (1000MID_50_) dose as these were all below detection limit.

**Figure 4:**
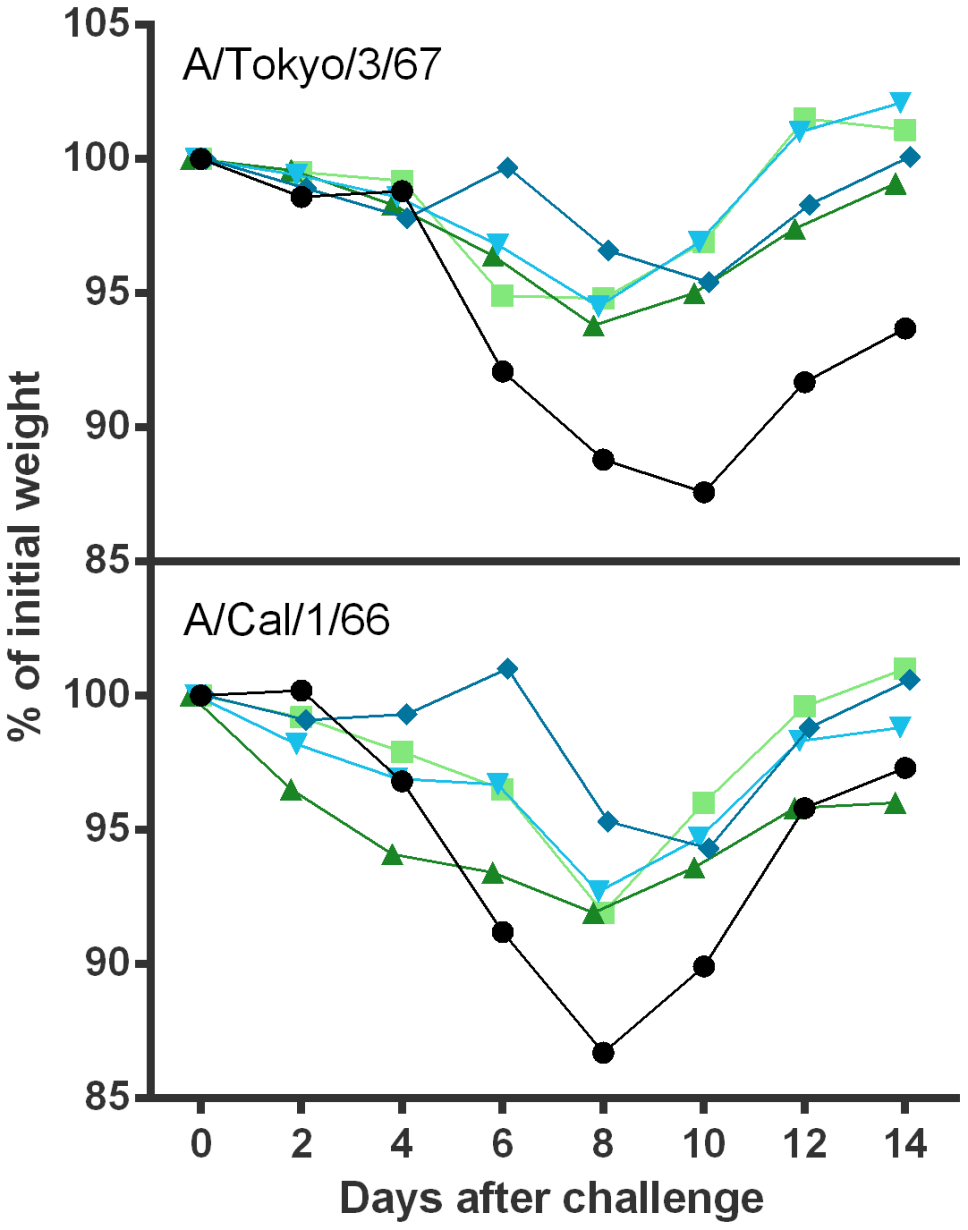
Percentage weight loss of mice challenged with H2N2 wild-type viruses. Animals were vaccinated twice with 17/Cal/395 (blue) using a low dose (diamonds) or a high dose (inverted triangles) or with 17/Tok/326 (green) using a low dose (triangles) or a high dose (squares). Placebo animals are indicated by circles. The animals were challenged 21 days after second vaccination with 100 MID_50_ of either A/California/1/66 (lower panel) or A/Tokyo/3/67 (upper panel) wild-type virus on day 42 after the first immunization. Five mice in each group were weighed and monitored daily until 14 days after challenge.

It is important to note that despite the variations in levels of serum HI antibodies induced by H2N2 LAIV candidates, all immunized mice were fully protected from subsequent challenge with both homologous and heterologous wild-type H2N2 viruses. Figure 3 demonstrates that all mice, which received two doses of either H2N2 LAIV vaccine were protected from wild-type virus replication in respiratory organs. In contrast, control mice shed virus in the lungs and in the nasal turbinates at high levels of 3.9–5.8 log_10_ and 2.2–3.2 log_10_ EID_50_, respectively. Neither in mock nor in vaccinated mice, virus was detected outside the respiratory tract (spleen and brain). Mock-immunized mice showed clinical signs of intoxication (rapid breathing, decline in motor activity, loss of appetite), which was accompanied by 15–17% weight loss by day 8 to 10 after challenge with either of the wild type viruses (Figure 4). In contrast, vaccinated mice did not exhibit clinical signs of infection and maximal weight loss did not exceed 8% (Figure 4). These findings indicate that a two dose immunization with either H2N2 LAIV candidate is sufficient to protect mice from challenge with homologous and heterologous wild-type H2N2 influenza viruses.

#### 3.4.3 Immunogenicity and protective efficacy of H2N2 LAIV reassortants in a ferret model

**Figure 5:**
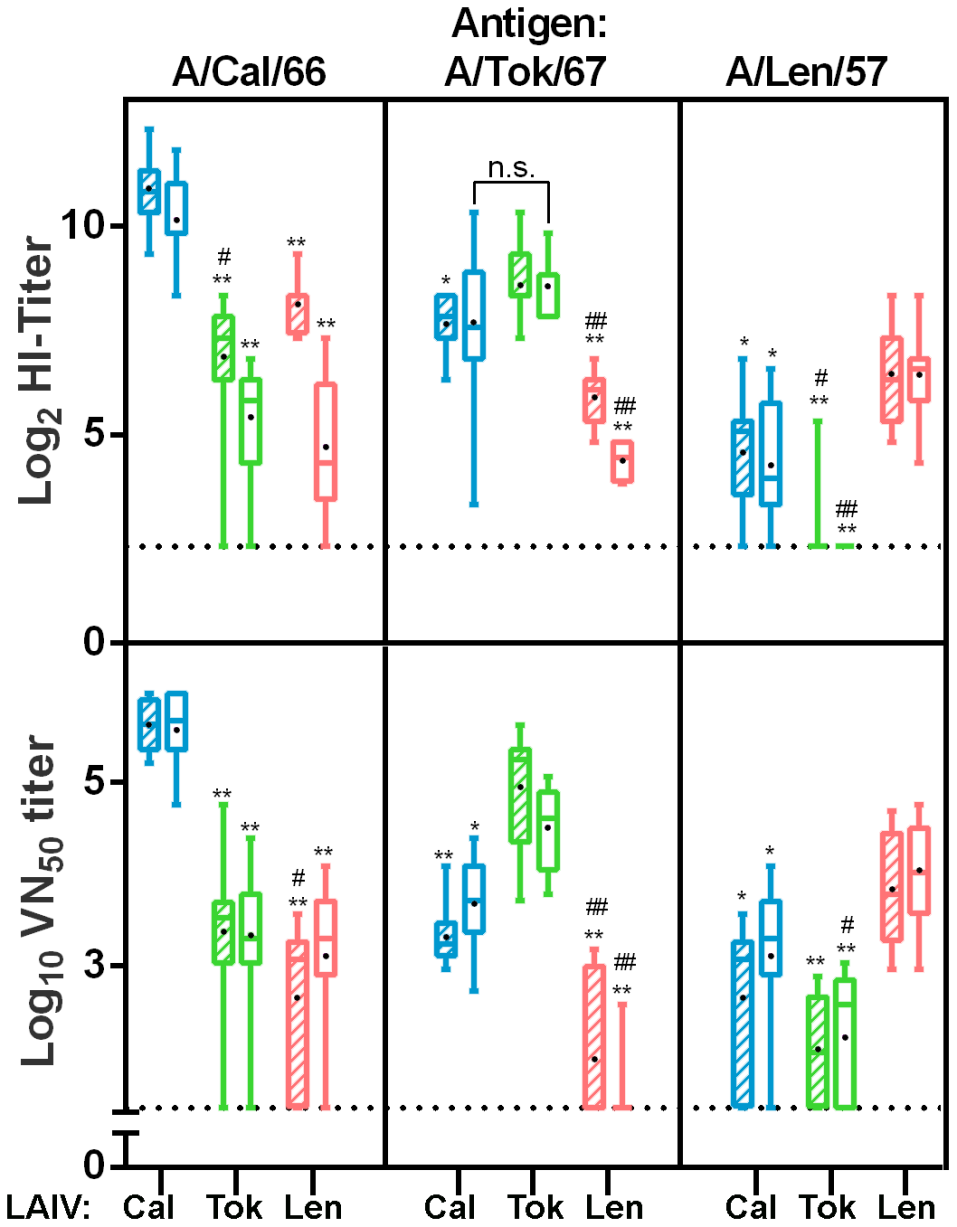
Haemagglutination Inhibition and Virus Neutralization titers in ferrets. Homologous and cross-reactive antibody responses in ferrets 14 (hatched boxes) and 21 (open boxes) days after vaccination represented by HI-titers (upper panel) and VN_50_ titers (lower panel). Titers induced by the 17/Cal/395 (blue), 17/Tok/326 (green) and Len/17 (red) vaccines are grouped by the respective antigens indicated at the top of the panels. Titers were measured against the vaccine strain as a representative of haemagglutinin of the wild type viruses (HI-titer determination) and against the wild-type strains (VN_50_ determination). Serum was titrated against 4HAU of the respective virus using 0.5% turkey erythrocytes (HI-titer determination) and against 100 TCID_50_ of the respective wild type virus (VN_50_ determination). In sera of placebo-vaccinated animals and sera prior to vaccination, no titers above detection limit were detected (not shown). Presented are box plots of log_2_ transformed HI-titers and log_10_ transformed 50% Virus Neutralization titers. Bars indicate the 5–95 percentile and black dots indicate the average titer. Significant lower titers with respect to the homologous titers are indicated by * (p<0,05) or ** (p<0,0001) and significant lower titers compared with the other heterologous strain are indicated by ^#^ (p<0,05) or ^##^ (p<0,0001).

Female ferrets (12 per group) were vaccinated intranasally once with either the 17/Cal/395 or 17/Tok/326 LAIV strain or a placebo. In addition, the master donor virus Len/17 was included as a control vaccine virus. Homologous and cross reactive antibody responses were assessed by haemagglutination inhibition and virus neutralization using the A/Cal/1/66, A/Tok/3/67 and A/Len/134/57 viruses or their respective vaccine strains as antigens. As shown in Figure 5, all three cold-adapted strains induced high homologous HI and VN antibody titers as was detected in sera taken 14 and 21 days after vaccination. The 17/Cal/395 strain induced the highest homologous titers, followed by 17/Tok/326 and Len/17, respectively. In most cases, the LAIVs also induced HI and VN titers against their heterologous strains. Strikingly, 17/Tok/326 did not induce cross reactive HI-antibodies against the earlier strain (A/Len/134/57); however virus neutralizing titers were detected, indicating other antibodies than those directed against HA play a role. The 17/Cal/395 LAIV strain induced cross reactive antibody titers that were only slightly lower than and in one case not significantly different from the homologous strains (Figure 5). In contrast, cross-reactive antibody titers induced by 17/Tok/326 and Len/17 were a couple of logs and significantly lower (or absent) than the titers induced by the homologous strains and also lower than the heterologous 17/Cal/395 LAIV titers. Furthermore, over time the cross-reactive responses seemed to decline for Len/17 and were more robust for 17/Cal/395.

**Figure 6:**
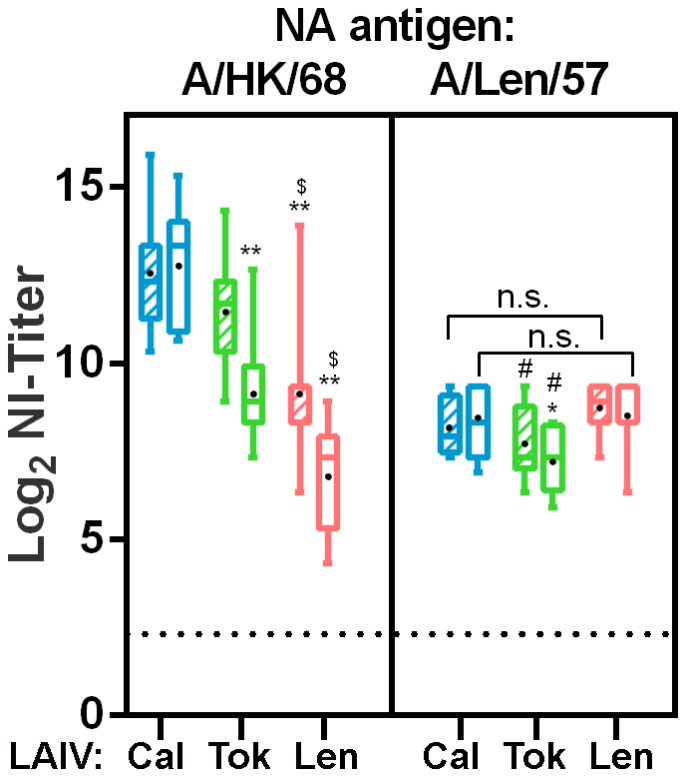
Neuraminidase Inhibition antibody titers in ferrets. Antibody responses in ferrets against early (1957) and late (1968) Neuraminidase 14 (hatched boxes) and 21 (open boxes) days after vaccination. Titers induced by the 17/Cal/395 (blue), 17/Tok/326 (green) and Len/17 (red) vaccines are grouped by the respective antigens indicated at the top of the panels. The X31 strain that contains the HA and NA genes of A/Honk Kong/1/1968 (H3N2) was used as a representative for the late NA genes, whereas the reassortant A/17/duck/Potsdam/86/92 (H5N2), which contains the N2 of A/Leningrad/134/1957 (H2N2) was used as a representative of the early NA genes. NI-titers were defined at 50% inhibition of the total neuraminidase activity of a standardized amount of the viruses. In sera of placebo-vaccinated animals and in sera taken prior to vaccination, background titers were detected, but were considered aspecific effects of the serum (not shown). Presented are box plots of log_2_ transformed NI-titers. Bars indicate the 5–95 percentile and black dots indicate the average titer. Significant lower titers with respect to the 17/Cal/395 induced NI-titers are indicated by *. Significant lower titers with respect to the 17/Tok/326 induced NI-titers are indicated by an ^$^ and significant lower titers with respect to the Len/17 induced NI-titers are indicated by an ^#^. One symbol refers to p<0.05 and two symbols refer to p<0.0001.

We next analyzed the ferret sera for the presence of (cross-reactive) neuraminidase antibodies in an assay in which the antibody dependent inhibition of desialysation of fetuin by a standardized amount of virus associated neuraminidase activity can be measured. A restriction of this assay is that viruses that share similar HA antigens as the vaccine strain cannot be used since antibodies in serum to HA may also inhibit neuraminidase activity through steric hindrance. We therefore used the HK-X31 strain, a PR8 based reassortant containing the HA and NA genes of A/Hong Kong/1/1968 (H3N2) as a representative of the late neuraminidase proteins. As a representative of the early NA genes, the Len/17 based A/17/duck/Potsdam/86/92 (H5N2) reassortant, which contains the N2 gene of A/Leningrad/134/1957 (H2N2) was used. The A/Cal/395 vaccine induced significantly higher NI titers than A/Tok/326 and Len/17 against late NA (Figure 6). With respect to the cross-reactive capacity of these antibodies, similar titers against the early NA as the titers induced by homologous Len/17 were detected. Vaccination with A/Tok/326 also resulted in cross-reactive antibodies against the early NA but these were significantly lower than the titers induces by Len/17.

To evaluate the protective efficacy of the H2N2 LAIV candidates, 21 days after vaccination six animals of each group were intranasally challenged with A/Cal/1/66 wt virus and the remaining six ferrets with A/Tok/3/67 wt virus at a dose of 10^6^ TCID_50_. In prior titration experiments, infection with wild type H2N2 viruses induced only a mild disease and did not induce significant clinical signs such as nasal discharge and sneezing and body weight loss (data not shown). However, in placebo vaccinated animals, fever peaks were recorded during the first three days after challenge by implanted temperature loggers. Fever was more pronounced after challenge with A/Cal/1/66 than after challenge with A/Tok/3/67. All vaccinated animals showed a reduction in fever after challenge with either of the H2N2 wt viruses compared to fever recorded in placebo recipients. The temperature increase over the course of the challenge phase (area under curve) was higher in the placebo-vaccinated animals than in the LAIV vaccinated animals, but only significantly different in the A/Cal/1/66 challenge groups (Table 4). The maximum temperature recorded after challenge was approximately 1°C higher in the placebo groups than in the LAIV vaccinated groups, but only significantly different in the A/Cal/1/66-challenged animals.

**Table 4:** Analysis of the body temperature during five days after challenge of ferrets immunized with H2N2 LAIV strains.

Vaccine group	Area Under Curve^1^ Day 21–26			Max delta T^2^ Day 21–26		
	Average	SD	N^3^	Average	SD	N^3^
A/Tokyo/3/67 challenge						
Placebo	1.4	0.5	2	2.4	0.6	2
17/Cal/395	0.2	0.3	4	1.5	0.4	4
17/Tok/326	0.1	0.2	4	1.7	0.2	4
Len/17	0.1	0.5	4	1.4	0.6	4
A/California/1/66 challenge						
Placebo	2.5	1.1	4	2.4	1.3	4
17/Cal/395	0*	1	5	1.6*	0.2	5
17/Tok/326	−0.7*	0.6	3	1.5*	0.3	3
Len/17	0.5*	0.4	5	1.4*	0.3	5

1 The Area Under Curve was deduced from the area under curve (after challenge) above the baseline (38.0 ± 0.7 °C) temperature (before challenge);

2 The maximum temperature increase represents the highest recorded increase in temperature above the baseline during any of the measurements after challenge;

3 Number of the temperature loggers used for the calculations. Initially all ferrets we implanted with a transponder, but after recovery the measurements of some of the transponders could not be retrieved, due to battery or recording failure;

*indicates significant difference compared to placebo group (p<0.05).

The body temperature was recorded at intervals of 30 minutes by an intraperitoneally implanted temperature transponder.

**Figure 7:**
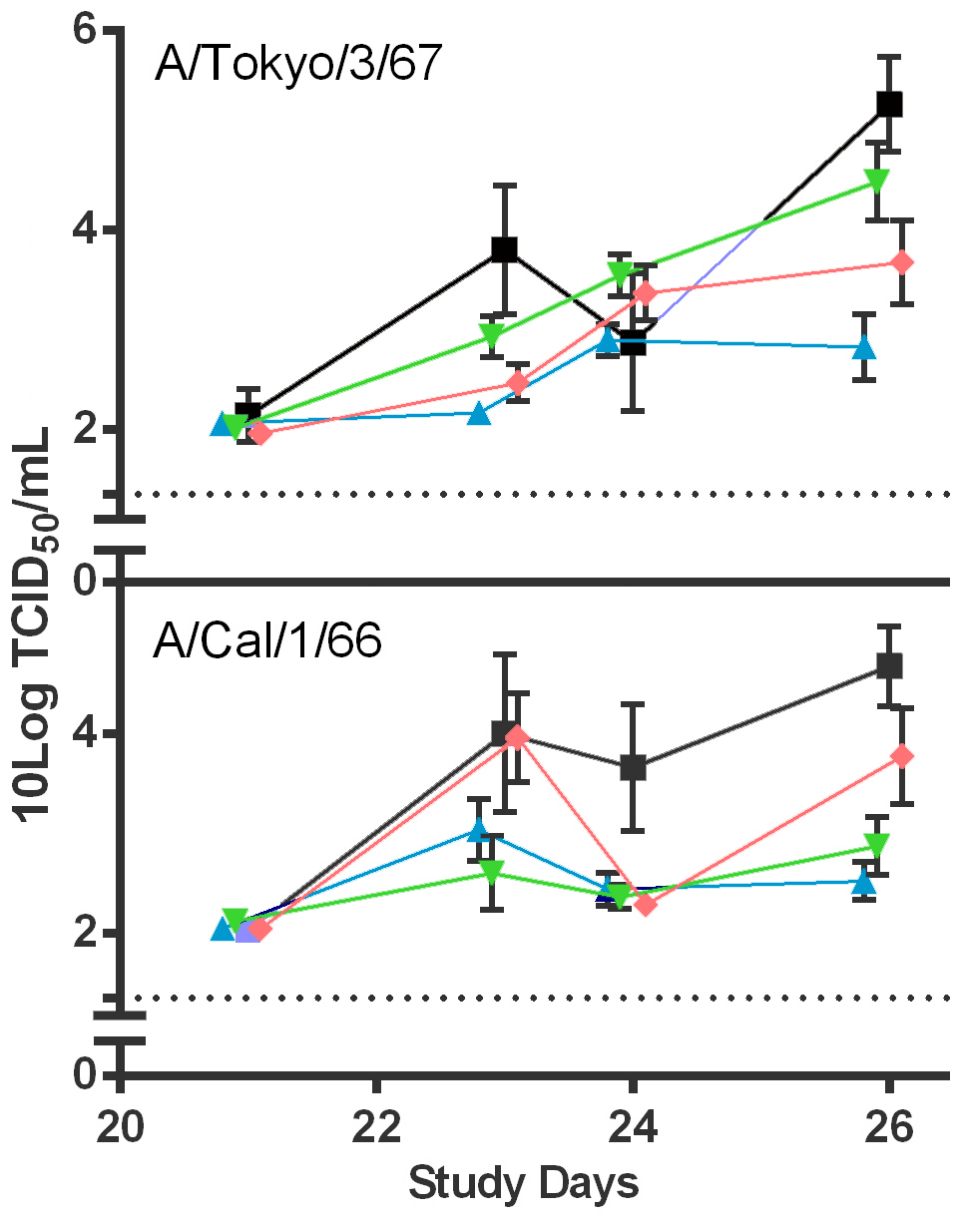
Virus titers in the throat swabs of ferrets challenged with wt H2N2 viruses. Ferrets vaccinated with either placebo (squares), 17/Cal/395 (triangles), 17/Tok/326 (inverted triangles) or Len/17 (diamonds) were challenged 21 days after vaccination with either A/Tok/67 wt (upper panel) or A/Cal/66 wt (lower panel) virus. Throat swabs were taken prior to the challenge and 2, 3 and 5 days after challenge. The virus titer (TCID_50_) in the transport buffer was determined by end-point titration on MDCK cells using a 5 fold serial dilution. Presented are the average log_10_ transformed titers. Bars represent the standard deviation and the dotted line indicates limit of detection.

Virus titers detected in the throat swabs taken after challenge increased until 5 days after the infection in the placebo-vaccinated animals (Figure 7). In general, virus replication tended to level in the LAIV vaccinated animals at 2 (A/Cal/66) and 3 (A/Tok/67) days after challenge and were lower than in the placebo animals. Strikingly, 17/Tok/326 LAIV provided the least protection against homologous challenge with A/Tok/3/67wt, whereas 17/Cal/395 protected better against this heterologous strain followed by the Len/17 MDV. After challenge with A/Cal/1/66, the homologous 17/Cal/395 and heterologous 17/Tok/326 provided equal protection against virus replication and clearly reduced virus titers were observed in respective vaccinated groups. In this respect, the Len/17 performed less well.

**Figure 8:**
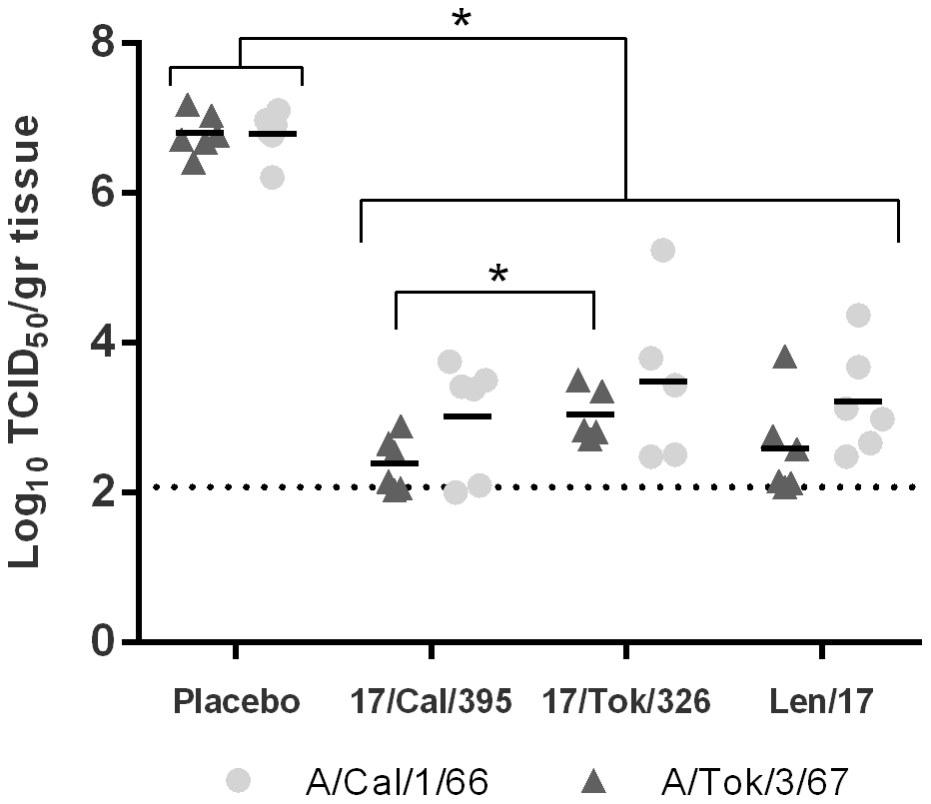
Virus titers in the nose. Virus titers in the nose (nasal turbinates) 5 days after challenge with either A/Tokyo/3/67 (triangles) or A/California/1/66 (circles). The virus titers in the homogenized samples were determined by end-point titration on MDCK cells using a 5 fold serial dilution. Presented are the individual log_10_ transformed titers and the average is presented by a bar. * indicates a significant difference (p≤0.05)and dotted line indicates limit of detection.

Five days after challenge (day 26), ferrets were sacrificed and tissues of the respiratory tract were isolated for determination of virus titers. In the nasal turbinates of the placebo-vaccinated animals, high virus titers were detected after infection with either of the H2N2 wt viruses (Figure 8). All the LAIV vaccinated animals showed a significant reduction of virus replication in the nose either for the homologous or heterologous challenge strains. In some animals of the 17/Cal/395 and the Len/17 vaccinated groups, virus replication was below the detection limit. Moreover, the 17/Cal/395 LAIV protected significantly better against virus replication of the heterologous A/Tok/3/67 wt strain than homologous 17/Tok/326 LAIV. This picture is consistent with the titers found in the throat swabs. Virus replication was not detected in the lower respiratory tract (trachea and lung), neither in the placebo-vaccinated nor in the LAIV vaccinated groups.

**Figure 9:**
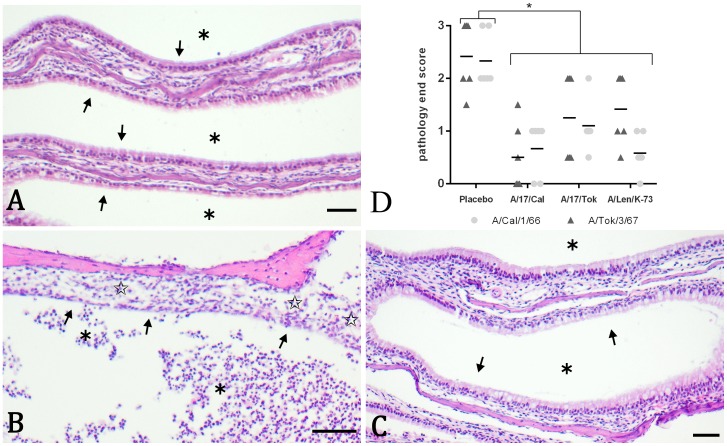
Histopathological representation and analysis of infection damage. Representative protection against damage induced by H2N2 wt infection in the nasal turbinates 5 days after challenge (shown here: A/Cal/1/66). Represented are a negative control ferret that was not vaccinated and challenged with a placebo (Panel A), a placebo-vaccinated animal challenged with wt virus (panel B) and an LAIV (in this case 17/Tok/326) vaccinated animal challenged with wt virus (panel C). Shown are 5 µm sections stained with haematoxylin and eosin with bars indicating 50 µm. Stars indicate moderate exudate of polymorphic nuclear cells in the lumen (panel B) or absence of exudate (panel A and C). Arrows indicate flattening or absence of the epithelial lining (panel B) or normal epithelial lining (panel A and C). Open stars indicate presence of inflammation in the submucosa (panel B) or absence/reduced inflammation and normal epithelium (panel A and C). The infection-induced pathology was scored on a scale of 1–5 based on the parameters described in the materials and methods in which a score of 0 refers to no aberration and a score of 5 refers to severe aberrations. In panel D, the overall infection pathology (end score) after challenge with either A/Tokyo/3/67 (triangles) or A/California/1/66 (circles) is presented. *indicates a significant difference (p≤0.05).

Intranasal challenge with either of the wild type challenge strains induced similar and clear infection damage in the nasal turbinates, but not in the trachea and lung. Damage to the epithelial lining of the turbinates in placebo-vaccinated ferrets was restricted to the respiratory epithelium and was absent in the olfactory epithelium. The respiratory epithelium showed loss of cilia, hypertrophy of goblet cells, irregularities and sometimes pseudo stratification and flattening (Figure 9B). Sporadically the lining was absent. A minimal to slight inflammation of polymorph nuclear cells (PMN) in the submucosa and between the epithelial cells was present. Finally, a moderate amount of neutrophilic exudate in the lumen was observed. The aforementioned histopathology parameters were individually scored in a semi-quantitative manner on a scale of 0–5. From these scores and the percentage of tissue affected, an overall infection damage score (end score) was deduced. This approach resulted in an end score of 2.3 and 2.4 for placebo-vaccinated animals challenged with A/Cal/66 and A/Tok/67, respectively (Figure 9D). Animals vaccinated with either of the LAIVs showed absent to moderate pathology after challenge with either of the two H2N2 strains. The epithelial lining was still intact, the inflammation in the submucosa and between the epithelial cells and the exudate in the lumen was clearly reduced (Figure 9C) and showed a similar picture as in placebo challenged control animals (Figure 9A). The end score for all vaccinated animals was significantly lower when compared to the placebo-vaccinated animals (Figure 9D). No statistically significant differences were observed between the different vaccine groups for the end score.

Altogether, vaccination of ferrets with the two H2N2 LAIV candidates and subsequent challenge with the respective wild type viruses demonstrated better immunogenic and cross-protective potential of the 17/Cal/395 strain compared to the 17/Tok/326 vaccine virus.

#### 3.4.4 Neuraminidase activity of H2N2 LAIV reassortants

**Figure 10:**
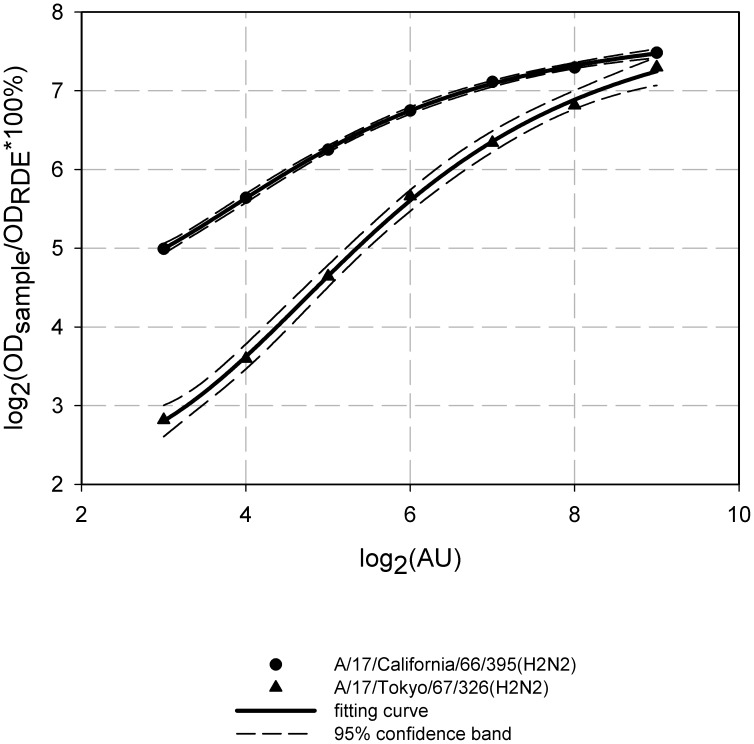
NA activity in percent of RDE activity for H2N2 LAIV candidates. NA activity was assessed in the fetuin cleavage assay. The analysis was performed at virus concentration of 8–512 agglutination units in triplicate and the average activity of strains tested was expressed as a percentage of positive control (RDE). Dashed lines indicate the 95% confidence band of fitting curve (continuous line).

To find possible reasons for the differences between 17/Cal/395 and 17/Tok/326 immunogenicity and protective efficacy, we compared NA enzymatic activity of the vaccine reassortants since this factor can possibly contribute to the strength and the width of virus-induced adaptive immune response due to the impact on virus spread in vaccinated individuals. Though our findings regarding virus growth in respiratory organs of infected mice don't support this assumption (17/Tok/326 had higher titers in lung than 17/Cal/395), nevertheless there is a possibility that the replication of 17/Cal/395 strain was more prolonged than that of 17/Tok/326, and additional experiments on kinetics of virus replication in mice are needed. Figure 10 represents the dependence of sialidase reaction product release on virus concentration in agglutination units. Interestingly, NA-induced desialysation of fetuin caused by 17/Cal/395 strain was significantly more effective compared to equal dose of 17/Tok/326 virus. These findings may indicate that either NA activity of 17/Cal/395 is higher than enzymatic activity of 17/Tok/326 or that these two strains differ by their HA/NA ratio.

In the first case, this could explain the higher homologous and cross reactive functional (HI, VN and NI) antibodies titers detected after 17/Cal/395 vaccination with respect to 17/Tok/326, since the virus would spread more efficiently and grow to higher titers. In the latter case, it would only explain the higher NI titers in 17/Cal/395 since NA density of this LAIV would then be higher.

## Discussion

Influenza A (H2N2) viruses disappeared from circulation among humans in 1968 but H2 subtype viruses continue to circulate in the avian reservoir which is confirmed by routine monitoring of wild migrating birds and poultry [7]–[9], [22]–[27]. Genetic analyses of 1957 pandemic H2N2 virus revealed that HA, NA and PB1 genes were derived from avian H2 virus and the remaining five genes were from contemporary H1N1 human influenza virus [28], [29]. During the early stage of H2N2 virus circulation, its receptor-binding specificity easily shifted from α2,3 avian-type to α2,6 human-type resulting in readily transmissible virus causing widespread and sustained disease in humans [30], [31]. Therefore, with declining ratio of anti-H2 immunity in the human population, the chances for H2 subtype viruses to cross the interspecies barrier again are growing over time posing a threat of a new pandemic. Recent isolation of H2N3 virus from pigs with respiratory disease in North America provides additional evidence for the ability of direct infection of mammals by avian H2 subtype viruses [11]. Therefore H2 influenza viruses are considered potentially pandemic and have been included into the WHO Global Influenza Preparedness Plan which strives for establishment of National and International Collections of vaccine strains against potentially pandemic influenza to promptly start a vaccination campaign when a pandemic breaks out [32].

Two major types of influenza vaccines for pandemic preparedness are widely used – inactivated influenza vaccine (IIV) and live attenuated influenza vaccine (LAIV). IIVs are injected parenterally and mostly induce humoral antibodies which are strain-specific. In contrast, LAIV administered via nasal spray induce a much broader immune response and can protect against drifted variants of influenza viruses [33]–[39]. In addition, LAIV can induce cytotoxic CD8+ T-cells directed against epitopes that are highly conserved throughout most influenza A subtypes and therefore may confer heterosubtypic protection [40]–[45]. All these advantages contributed to the inclusion of LAIVs into the WHO global influenza preparedness plan [32].

To date several H2N2 cold-adapted vaccine candidates have already been evaluated. Two of them are used as master donor viruses for preparing LAIVs in Russia and the USA: A/Leningrad/134/17/57 and caA/Ann Arbor/6/60 (caA/AA), respectively. Len/17 MDV was used for immunization in the 1960-s and was proven to be safe and immunogenic in children and adults [12], [46]. In addition, this strain was evaluated in animal models and was shown to be a superior immunogen compared to the more attenuated strain A/Leningrad/134/47/57 and caA/AA virus, on the basis of significantly higher IgG and IgA responses in mouse lung [20]. Moreover, in this study, we show that the Len/17 strain, which was included to assess the cross-protective effect of this well-characterized master donor virus induced (cross) reactive VN, HI and NI antibodies in ferrets and protected against heterologous challenge with A/Cal/66 and A/Tok/67. Since many of recently isolated avian H2 viruses are antigenically close to 1957 H2N2 pandemic virus, it is reasonable to suggest protective potential of Len/17 MDV against such viruses, should they re-emerge in humans [9]. Indeed, Chen et al. tested a number of avian and human H2 influenza viruses and demonstrated great cross-reactivity of mouse and ferret sera raised against early human virus Japan/57, which in known to be genetically and antigenically close to the Len/17 MDV, with currently circulating avian H2 influenza viruses [47]. Therefore, Len/17 is a good candidate for pandemic H2N2 LAIV with no further pre-clinical and clinical evaluation required. caA/AA virus, which does not have a vaccine history like Len/17 demonstrated safety, immunogenicity and (cross) protective efficacy in mice and ferrets [48]. Surprisingly, a clinical study showed that the vaccine was restricted in replication and was minimally immunogenic in healthy adults born after 1968 [49]. The authors proposed that extensive passaging of caA/AA virus in CEF cells and eggs over the past few decades decreased the ability of the virus to replicate in humans, and that low passage human virus isolates should be used as HA and NA source for H2N2 LAIV [49].

This strategy was used in our study: we prepared H2N2 LAIV reassortant candidates from human H2N2 viruses isolated at the end of H2N2 cycle. The rationale for choosing human viruses rather than currently circulating avian H2 viruses was to allow better replication of vaccine viruses in humans and therefore inducing stronger immune responses. H2 viruses of avian origin are more cross-reactive in animal models, but not able to replicate in humans and most probably won't induce even homologous immune response. Indeed, this trend was observed for vaccines against highly pathogenic avian H5N1 viruses which were highly immunogenic and cross-reactive in animal models but failed to induce strong immune responses in clinical trials [50]. We chose two representative strains from different H2 lineages which were evolutionary diverged and as a result, the viruses almost didn't cross-react in an HI test with immune rat sera (Table 3) [13]. Importantly, these H2N2 wild-type viruses had a low passage history and therefore should have retained their ability to infect humans.

We generated two 6∶2 LAIV reassortant viruses A/17/California/66/395 and A/17/Tokyo/67/326 using a modified reassortment technology, where intermediate H1N1 reassortant virus was used to deliver Len/17-specific internal genes into the genome of vaccine reassortant candidates. Safety of these strains was confirmed in *in vitro* studies demonstrating that both H2N2 LAIV candidates had *ts/ca* phenotype identical to that of MDV Len/17 and in previously performed toxicological studies in mice and guinea pigs showing absence of mortality, morbidity, weight loss, behavioral changes and histopathological lesions (data not shown). When an additional safety study in mice confirmed the attenuated phenotype of the candidates after intranasal inoculation, we further moved to the assessment of their immunogenicity and protective efficacy in mice and ferrets.

Remarkably, in both animal models 17/Cal/395 LAIV candidate demonstrated clear advantages over 17/Tok/326 strain in terms of induction of serum HI antibodies (in mice and ferrets) and VN antibodies (in ferrets only) to the homologous antigen (see Figures 2 and 5). However, when analyzing cross-reactive antibodies some discrepancies were observed between mouse and ferret models since 17/Tok/326–immunized mice showed better cross-reaction (Figure 2A), whereas in ferrets 17/Cal/395 induced better cross-reactive antibody responses when compared to 17/Tok/326 and Len/17 MDV (Figure 5). Such discrepancies between cross-reactivity data from these two animal models were noted previously for avian and human H2 subtype influenza viruses [47]. The authors noted variable degree of concordance between the use of ferret and mouse antisera in HI and neutralization assays. But nevertheless, among 15 viruses tested, they were able to identify three strains which induced broadly cross-reactive antibody response in mice and ferrets [47].

In addition to LAIV induced HI and VN antibodies in ferrets, we analyzed the induction of antibodies in ferrets that could inhibit the neuraminidase mediated cleavage of sialic acid from fetuin. These antibodies are being paid far less attention to than HI antibodies, which might be inapt, since a recent paper showed that NA antibodies correlated well with protection against lethal H5N1 infection when ferrets were immunized with a 2009 pandemic H1N1 vaccine [51]. The NI inhibition assay excludes the use of viruses with the same subtype as the vaccine strain, since HA antibodies could produce false positive inhibition through steric hindrance. We therefore used A/Hong Kong/1/1968 (X31) H3N2 as a representative of the late neuraminidase proteins and the A/17/duck/Potsdam/86/92 H5N2 reassortant which contains the N2 gene of pandemic A/Leningrad/134/1957 as a representative of the early NA genes. Sequence comparison showed that NA genes of 1968 H3N2 viruses were similar to NA of 1966 and 1967 H2N2 isolates belonging to clade I NA genes, which includes A/Cal/66 and A/Tok/67 [13]. Once more, the A/Cal/395 LAIV appeared superior, since the LAIV induced clearly higher NI antibodies against late and also early NA variants than A/Tok/326. In addition, the A/Cal/395 induced cross reactive NI antibody titers against the early NA were similar to those induced by the homologous MDV Len/17.

To further analyze which of the LAIV strains would have clear advantages over another, we conducted detailed analysis of their cross-protective efficacy in ferrets, since this model is considered the most appropriate for studying human influenza viruses [52], [53]. When placebo vaccinated ferrets were challenged with either A/Cal/1/66 wt or A/Tok/3/67 wt virus, they developed fever, but no clear clinical signs of influenza disease and weight loss was observed. High virus titers were detected in throat swabs and in the nasal turbinates but not in the lower respiratory tract. The infections induced a rhinitis in the nose, characterized by damaged respiratory epithelium, inflammation and neutrophilic exudate. In all the vaccinated animals fever was insignificant, virus replication was strongly reduced and the pathology in the nose was almost absent. Strikingly, in both the throat and nasal turbinates, reduction of A/Tok/3/67 virus replication was significantly higher for the heterologous 17/Cal/395 vaccinated animals than in the homologous 17/Tok/326 vaccinated animals and the infection damage in the nose was less for 17/Cal/395, although this was not significant. The MDV control Len/17 for which both challenge strains were heterologous, provided efficient protection; however the A/Cal/395 appeared superior. The higher HI, VN and NI homologous and heterologous antibody titers induced by the A/Cal/395 vaccine correlate well with this outcome. For this reason, the A/Cal/395 reassortant strain was chosen for submission to the Russian Ministry of Health for permission of a phase I clinical trial in healthy adult volunteers.

Our additional comparison of H2N2 LAIV candidates for their NA enzymatic activity revealed significant differences between the viruses and it will be interesting to investigate whether the higher activity of NA enzyme of 17/Cal/395 LAIV helps the virus to replicate better in the respiratory tract of immunized animals and therefore induce stronger and more cross-reactive immune responses.
